# The Influence of “Artificial Intelligence + Human–Computer Interaction” on Teachers’ Psychological Changes in Academic Management in Colleges

**DOI:** 10.3389/fpsyg.2021.730345

**Published:** 2021-11-19

**Authors:** Honghai Guan, Qingli Chen, Song Han, Baoge Zhang

**Affiliations:** ^1^Faculty of Education, Northeast Normal University, Changchun, China; ^2^School of Educational Science, Mudanjiang Normal University, Mudanjiang, China; ^3^Provincial Primary and Secondary School Teacher Development Center, Shaoguan University, Shaoguan, China; ^4^Department of Humanities and Social Sciences, Ningbo University, Ningbo, China

**Keywords:** artificial intelligence, human–computer interaction, colleges, academic management, psychological changes

## Abstract

The purpose was to analyze the psychological changes of teaching staff in the academic management of local colleges, and briefly explore the role of teaching staff in the development of the social economy and colleges. In the environment of artificial intelligence and human–computer interaction (HCI), first, the relevant theories of teaching staffs’ psychological status and the characteristics of teaching staff in college academic management were analyzed and expounded. Next, the way of the questionnaire was selected to analyze the psychology of teaching staff in college academic management at different ages, professional titles, academic qualifications, disciplines, and teaching years. The results showed that the mental health level of college teachers was lower than the current national adult standard; the mental health level of female teachers in colleges was higher than that of male teachers; the p value of mental health of college teachers with different ages, professional titles, education, disciplines, and teaching years was greater than 0.05, indicating that there was no significant difference; the *p*-value of professional academic and mental health was less than 0.01, indicating that there was a significant correlation, that was, teachers’ professional academic exerted a significant impact on teachers’ mental health. In short, under the background of artificial intelligence and HCI’s rapid development, higher education was moving forward with high quality, and more attention should be paid to the psychological changes of college teaching staff.

## Introduction

College teacher, as a knowledge-intensive and academic profession, bears a crucial responsibility for training high-quality talents for the country. Li (2019) thought that now there were too many things facing college teachers, not only teaching, still they have the same responsibility with primary and middle school teachers to train builders and successors for the motherland although there are some differences between them. The work of college teachers is a kind of complex mental work that differs from that of primary and middle school teachers (Li, 2019; Kholbutaevna, 2021). Colleges must undertake complex and onerous teaching tasks, as well as more difficult and creative academic tasks (Mccaffrey and Spector, 2018; Park et al., 2021). At present, a series of reforms in the overall education system and management system has been conducted in China’s higher education system to meet the needs of modern development, the internationalization of higher education, the concept of information-based learning and foreign advanced educational technology (Qian et al., 2018; Rogoza et al., 2018). Fischer (2020) thought that the researches on teacher creativity had focused more on values and resources and the reform not only provided a broad space and opportunities for the development of college teachers, but also brought great challenges to teachers.

There is an increasingly closer relationship between machines and humans with the development of technology and the arrival of the era of artificial intelligence (AI). Wang and Cheng (2019) thought that the man-machine relationship has also undergone corresponding development and changes with the gradual social characterization of intelligent machines with feedback functions such as man-machine dialogue, action, and emotion. The machine has developed from a functional tool attribute to a social role attribute. Bian et al. (2019) pointed out that the impact of intelligent machines on people is not only reflected in the changes of man-machine relations, but also reflected in the impact on people’s needs. Martínez et al. (2021) pointed out that, in general, it dealt with the design and development of machines and computers that best serves the user needs, and the intervention of AI leads to deeper interaction and greater uncertainty in the process of human–computer interaction (HCI). Colleges have more and more requirements, and teachers’ work tasks become heavier and heavier. This is because teachers face greater academic challenges, which affect their psychological status (Brusco et al., 2021). Ji (2020) thought that as a higher education group, college teachers have higher requirements for themselves. With the continuous development of teaching skills and technology, teachers must update their knowledge system and improve their learning and teaching ability, which also brings academic challenges to teachers (Colmenares, 2021). Special attention must be paid to the impact of teachers’ psychological status, teachers’ mental health level must be analyzed, and adaptation strategies must be explored to improve anti stress ability and teachers’ psychological status (Limniou et al., 2019).

For the research on teachers’ psychological changes in academic management in colleges, different researchers put forward different coping strategies from different angles. Regarding the research on the current situation and countermeasures of teachers’ mental health, Deng and Chen (2021) pointed out strengthening mental health education, paying attention to teachers’ mental health, respecting teachers’ reasonable needs, and establishing harmonious interpersonal relationships; besides, teachers should learn to adjust themselves. Chen (2019) put forward the countermeasures to solve the psychological problems of college teachers from the three levels of society, school, and individual. Monea (2020) emphasized how to alleviate teachers’ professional pressure and improve teachers’ mental health. Travers and Cooper believed that schools should not only train teachers how to engage in teaching and teach interpersonal skills, but also mobilize teachers’ sense of responsibility and interest in work. For school management, Carroll and Dahlstrom (2021) pointed out that schools should not blindly quote the management methods of enterprises due to their particularity.

First, the research progress in relevant fields and the shortcomings of existing theoretical research are realized through the research background and the research of existing relevant achievements in the world. Then, the existing important concepts and research background related to the subject are analyzed. Finally, in the AI and HCI environment, the questionnaire is selected to analyze the psychological change indicators of teaching staff. The research innovation is to combine AI and HCI with the psychological changes of college staff, in order to provide reference coping strategies for college staff in academic management (Deng et al., 2021).

Based on AI and HCI environment, questionnaire method is selected to analyze teachers’ psychological changes, so as to provide coping strategies for academic management and teaching personnel in colleges and universities.

First, the theoretical basis of academic management and psychological characteristics of college teaching staff is expounded. Then, the corresponding questionnaire is designed. Based on this method, the psychological changes of teachers in academic management in colleges are analyzed from multiple angles, and finally the corresponding conclusions are drawn. These conclusions and viewpoints are brand-new, which can be used for reference for subsequent related research.

## Analysis of Academic Management and Psychological Characteristics of Teaching Staff

### Artificial Intelligence and Human–Computer Interaction

Human–computer interaction and AI are two crucial research fields in the intelligent information age (Horgan et al., 2018). The relationship between them has changed with their development and typical applications based on their deep integration are also reflected in education (Schafer, 2019; Harré, 2021). HCI provides research ideas and application requirements for AI, while AI greatly promotes the transformation and development of HCI technology in the modern world (Vedapradha et al., 2019; Sharma, 2020). AI is a new technology and science for research and development to simulate, extend and expand human intelligence. It simulates human intelligence through machines, such as perception ability (visual perception, auditory perception, and tactile perception) and intelligent behavior (learning ability, memory and thinking ability, and reasoning and planning ability), so that machines can “think and act like people,” and finally machines can do the work that only talents could do in the past. The rapid development of AI has aroused heated discussion. Whether AI can replace people has become the focus of attention. As early as 1993, computer scientist Vernor Vinge put forward the concept of singularity, that is, AI driven computers or robots can design and improve themselves, or design more advanced AI than themselves. In the face of AI, people should not overestimate or underestimate it. It is essential to uphold a rational attitude toward the impact of AI on education. The main research fields of AI include intelligent control, natural language processing, pattern recognition, artificial neural network, machine learning, intelligent robot, and so on.

In the future, the two will maintain the current mutually promoting and driven relationship, and will further integrate and develop cooperation. Their integration has reached an unprecedented level (Villegas-Ch et al., 2021; Wang, 2021). The exploration of their integration method makes AI technology more persuasive and HCI technology more natural and practical (Wu et al., 2019; Wu and Song, 2019).

After two major delays in AI and HCI, people no longer imagine that computers are capable of surpassing human beings completely, which is impossible under current technical conditions. People turn to study the most practical and achievable issues, leading to the gradual division of AI into five relatively independent disciplines based on random probability model and calculation, namely, cognitive science, natural language understanding, computer vision, machine learning, and robotics (Zhang et al., 2021). AI and HCI are also reflected in the evaluation of psychological changes of college staff. Wu and Wu (2019) emphasized the application of computers in the study of psychological state.

### Related Theories of the Psychological Status of Teaching Staff

Teachers are a professional group different from other professions. The interpretation of their psychological status should be professional enough to reflect the particularity of their profession (Abascal et al., 2017). Hence, the connotation of teachers’ psychological status should include the psychological status of the general population, and reflect the particularity of teachers’ profession (Babarinde et al., 2021; Baeriswyl et al., 2021). The relationship between the teaching profession and other professions needs to be evaluated to reflect the particularity of teaching profession (Feng and Chen, 2020; Martínez et al., 2021).

The most prominent feature of the teaching profession is its special object of work – students, which determines the diversity of teachers’ responsibilities. Teachers should not only choose correct education methods and impart knowledge to students, but also exert a positive impact on students and promote their physical and mental development (Ibrahim, 2021). Moreover, teachers must meet the needs of students at different levels or groups (Bentil et al., 2020). The work achievement of teachers lags behind and is difficult to measure due to the particularity of the work object. Teachers’ mental health problems come from their own and external factors. According to the research of relevant literature, there are four kinds of mental health problems in higher vocational teachers, which are emotional problems, interpersonal problems, negative behavior problems, and personality obstacles. Emotional problems involve a wide range, including all bad emotions, so they are also the most common mental health problems among higher vocational teachers. The problem of interpersonal relationship requires that teachers and students must establish a good interpersonal relationship. Only in this way can they have a good communication with students and effectively transmit knowledge and learning methods to students; for negative behavior problems, teachers with mental health problems will have deviation in behavior, such as negative behavior in work and life.

The rapid development of China’s higher education provides teachers with broad space and opportunities for development. However, the comprehensive reform of the education system and management system also brings great challenges to teachers. Society has high expectations and requirements for higher education and teachers, and unprecedented vocational academic problems have emerged in the reform of higher education, resulting in some psychological problems of college teachers. The professional academic and psychological status of college teachers have a great practical significance for alleviating their professional academic and improving their psychological status (Treadway et al., 2020).

To sum up, AI and HCI gradually run through the academic management of college teaching staff.

### Characteristics of Teaching Staff in Academic Management of Colleges

College teacher is a highly educated group with an enterprising spirit and strong desire for creation. The academic pressure of higher education reform is unprecedented. Unbearable, excessive and long-term academic work will exert a certain negative impact on teachers’ physical and mental health, leading to an increase of the mental health problems of college teachers in recent years.

The media often report accidents that cause personal injury by extreme means such as suicide (Box, 2020; Gibbons et al., 2021). Thus, it is very urgent and crucial to focus on the professional academic and mental health of school teachers, analyze and study the origin of teachers’ mental health problems, and explore the adaptation strategies to maintain teachers’ physical health. The methods of mental health evaluation include self-evaluation, psychological test, pathological evaluation and classification, social adaptability standard, and so on. Common psychological problems are as follows: (1) Depression: it refers to a person’s long-term depression; (2) Paranoia: it often comes from unreasonable ideas about self and the outside world, or even delusions, such as the delusion of being loved, killed, jealous, and worshipped; and (3) Inferiority: people with inferiority tend to have a low evaluation of themselves, and may have other negative emotions or psychological problems, such as depression, excessive shyness, and so on; besides, anxiety, hypersensitivity, jealousy, and cruelty are also common psychological problems.

The psychological status of teaching staff is affected by multiple factors, including teachers’ personality, cognitive style, interpersonal relationship, coping strategies, and emotions. This exploration focuses on the application of AI in the academic management of teaching staff. The psychological changes of teaching staff with different ages, education backgrounds, professional titles, disciplines, and teaching years are evaluated. This exploration is based on AI and artificial interactive algorithm to analyze the psychological changes of teaching staff in academic management of colleges in many aspects. Compared with the studies cited, the data research degree of this exploration is deeper and wider, and some new conclusions are found, which has far-reaching significance for the research on the mental health problems of academic management staff in colleges.

## Questionnaire Design and Model Selection

### Questionnaire Design

Stratified random sampling is adopted to select teachers from 15 faculties of a college. A total of 300 questionnaires are distributed, and 252 valid questionnaires are finally obtained. The questionnaire consists of teachers’ basic personal information, including gender, age, discipline category, culture category, and education level. This questionnaire is made and issued on Questionnaire Star to collect data. Invalid questionnaires are eliminated, and SPSS25.0 mathematical statistics software is employed to analyze the data of effective questionnaires. The questionnaire has been approved by the participants. It is an anonymous survey for scientific research, and does not involve ethical factors, so the relevant data obtained are strictly confidential.

### Research Tool

SCL-90 is a mental health self-evaluation scale that has been widely used and accepted by Derogatis (1975). It includes 90 evaluation elements with a wider range of content, such as feeling, emotion, sleep, consciousness, and thought. Ten factors, namely somatization (A), compulsion (B), interpersonal sensitivity (C), depression (D), anxiety (E), hostility (F), terror (G), paranoia (H), psychosis (I) and other (J), are adopted to reflect the 10 aspects of psychological symptoms.

Starting with discriminant analysis, first, the total score of each element is calculated. Then, the data are rearranged from top to bottom according to the total score, and 25% of the subjects are divided into high group and low group, respectively. Then, the *T*-test of independent samples is used to test the difference of subjects’ scores on each question, which can compare whether the difference between the two averages is significant. Besides, some elements are deleted according to whether the element limit ratio reaches a meaningful judgment standard. Next, the higher the standard deviation is, the greater the difference between individuals is and the wider the data distribution is. On the contrary, the smaller the distribution range of individual scores is, the smaller the difference of individual response is. Finally, it is essential to determine the factor analysis, properly test the data and generate the final results. Shen et al. (2019) analyzed social behavior and proposed concept link mining method for analysis, which strongly confirmed AI’s research on psychological changes of university staff.

### Questionnaire

Table 1 displays the basic demographic characteristics of the respondents.

**TABLE 1:** Statistics of demographic data of the investigated teaching staff.

	Gender		Age			Subject category		Educational level		
Basic information	Male	Female	<35 years old	35–50 years old	>50 years old	Science departments	Liberal arts	Undergraduate	Master	Doctor
Number of students	138	114	79	141	32	151	101	85	79	88
Percentage (%)	54.8	45.2	31.3	56.1	12.6	59.9	40.1	33.7	31.3	35

The feasibility of the scale is tested, and Table 2 displays the results. Fifty subjects are randomly tested. The data of the prediction scale are tested by discrimination test, standard deviation test, and exploratory factor analysis. Then, some invalid items are deleted to form a formal scale.

**TABLE 2:** Test of statistics and sphericity.

Measure	Approximate chi-square	DF	Sig
0.894	9300	345	0.000

*Sig represents the level of significance.*

In Table 2, the sphericity test of teacher academic scale is 9299.812, with a high value, and the corresponding probability is 0.000, which is very significant, indicating that there may be sharing factors among elements. Meanwhile, the test statistic value is 0.894, showing that the data is suitable for factor analysis. Exploratory factor analysis is conducted on the teacher academic scale. The scale is extracted by principal component analysis, and the results are rotated orthogonally to the maximum extent.

In exploratory factor analysis, the data are first tested for fitness to test whether they are suitable for factor analysis. Table 3 presents the test results.

**TABLE 3:** Reliability analysis.

	Compulsion	Somatization	Interpersonal sensitivity	Depression	Anxiety	Hostility	Terror	Paranoia	Other	General table
Cronbach coefficient	0.757	0.754	0.753	0.766	0.788	0.765	0.789	0.765	0.768	0.754

The scale is evaluated by the internal consistency reliability analysis of each factor. As shown in Table 3, the common factors and the Cronbach coefficients of the total table are 0.757, 0.754, 0.753, 0.766, 0.788, 0.765, 0.789, 0.765, 0.768, 0.754, and 0.757, respectively. The results show that the questionnaire has good reliability. The Cronbach coefficients of the three factors and the total table are all above 0.7, which are acceptable, indicating good credibility of the questionnaire.

## Discussion on Questionnaire Test Results

### The Overall Reflection of Teachers’ Psychological Condition

**FIGURE 1:**
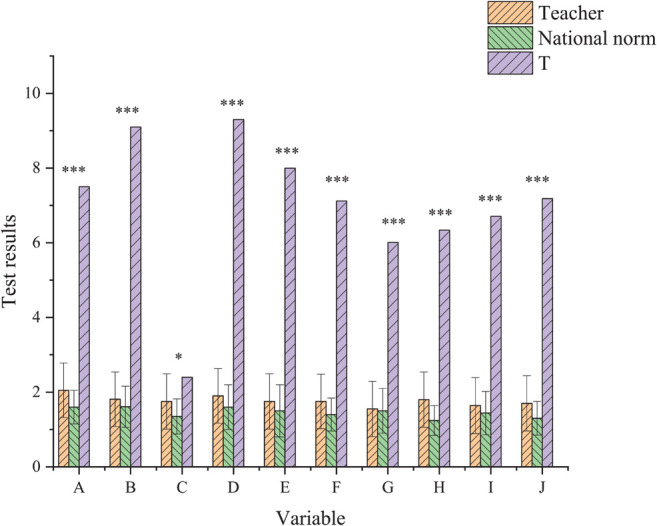
Comparison of the survey results of college teachers with the national norm (^∗^*p* < 0.05, ^∗∗∗^*p* < 0.001).

Unlike the national adult standard (Figure 1), the scores of SCL-90 among 252 college teachers are higher. Besides, the scores of the factors sensitive to interpersonal relationships are significantly higher than the national standard, and the *p*-value is less than 0.001.

One of the reasons is that the management system, which is formed by the internal fierce competition among the college teachers with very complex mental work, has been reformed. It makes the academic level of teachers’ profession in colleges higher than that in other industries, thus leading to the psychological academic level of teachers, and affecting the level of teachers’ mental health.

Then, the existing national adult standards were formulated in the late 1980s, when the social competition was much smaller than today. It is doubtful whether the normative norms still represent the present mental health level of adults in China, which is one of the reasons why the measurement results differ greatly from the national norms.

### Comparison of the Psychological Status of Teachers With Different Genders

**FIGURE 2:**
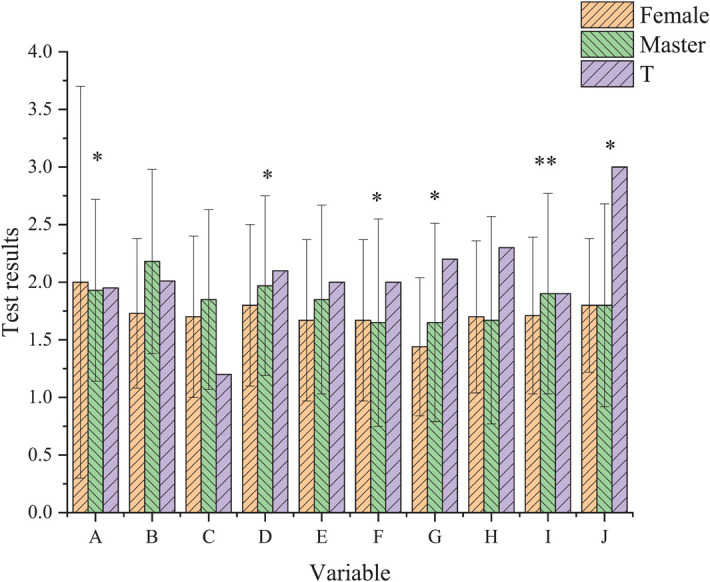
Comparison of survey results of male and female college teachers (^∗^*p* < 0.05, ^∗∗^*p* < 0.01).

The results of SCL-90 (Figure 2) suggest that male teachers have higher factor scores than female teachers. There are significant differences in somatization, depression, hostility, fear, and other psychological problems between male and female teachers, with *p*-value less than 0.05. There are significant differences in mental symptoms, with *p*-value less than 0.01.

Figure 2 displays the comparison of male and female teachers’ psychological status in SCL-90 results. The psychological level of male teachers is lower than that of female teachers. Their level of somatization, depression, hostility, terror, and other aspects are obviously lower than that of female teachers. Especially, their level of psychiatric symptoms is very low.

The social needs of females are often lower than that of male teachers due to their different roles. Male teachers should not only take care of their children and the elderly, but also undertake complex tasks such as leadership, teaching, and academic research. Male teachers tend to take on more responsibilities at work than female teachers, and they are more eager to obtain a sense of achievement in their profession. This strong desire itself will undoubtedly bring psychological pressure to male teachers.

### Comparison of the Psychological Status of Teachers of Different Ages

**FIGURE 3:**
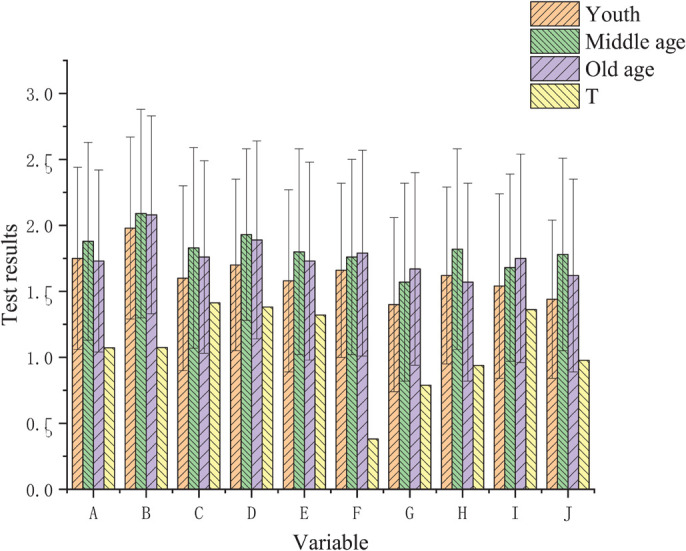
Comparison of survey results of teachers in different age groups.

College teachers are categorized into three age groups: young group, middle-aged group, and old group. The results of the SCL-90 test (Figure 3) illustrate that the scores of hostile factors of middle-aged and old teachers are higher than those of young teachers. However, there is no significant difference in the scores of different groups of teachers, because the *p*-value is greater than 0.05.

The survey shows that there is no significant difference in the psychological status of teachers in different age groups, which may be due to the different academic situations faced by teachers of different ages. The experience of young teachers is less, and the practical problems in the management of old and middle-aged teachers are less; young teachers shoulder more psychological academic work in teaching and research; the difficulties they meet promote the academic title; the life of young teachers is the heaviest, and their burden is higher than that of old and middle-aged teachers, especially those facing serious housing problems.

Nowadays, the gap between housing price and wage income, as well as the reform of housing and medical policies, bring special academics to young teachers who have no economic basis to solve basic problems such as housing. Middle-aged teachers should shoulder academic research and teaching, and look after the old and the children in family life; with the strengthening of higher education reform, middle-aged and old college teachers in China focus more on the stability of employment. The knowledge structure and the old teachers’ education mode no longer meet the current higher education needs due to the advent of the information age and explosive growth of knowledge; the spirit of retired teachers and their best efforts is conducive to their mental state.

### Comparison of the Psychological Status of Teachers With Different Educational Backgrounds

The qualifications of college teachers are classified into three groups of bachelor’s degree, master’s degree, and doctor’s degree. The results of SCL-90 (Table 4) show that the scores of most symptoms, such as depression, anxiety, and other factors of teachers with a doctoral degree, are higher than those of other teachers with a bachelor degree, while their scores of somatization factor, hostility, and paranoid degree are higher than those of teachers with bachelor’s degree of the same level. However, there is no significant difference in the scores of different disciplines.

**TABLE 4:** Comparison of survey results of teachers with different educational backgrounds.

	Somatization	Obsession	Interpersonal sensitivity	Depression	Anxiety	Hostility	Terror	Paranoia	Other
Undergraduate	1.86 ± 0.78	1.75 ± 0.76	1.87 ± 0.76	1.76 ± 0.81	1.78 ± 0.91	1.52 ± 0.77	1.80 ± 0.80	1.77 ± 0.80	1.78 ± 0.37
Master	1.74 ± 0.67	2.03 ± 0.72	1.75 ± 0.70	1.86 ± 0.75	1.70 ± 0.71	1.73 ± 0.71	1.50 ± 0.68	1.72 ± 0.71	1.60 ± 0.73
Doctor	1.90 ± 0.70	2.3 ± 1.82	1.87 ± 0.74	1.99 ± 0.71	1.82 ± 0.76	1.80 ± 0.71	1.56 ± 0.74	1.79 ± 0.73	1.68 ± 0.70
*F*	0.50	0.77	0.40	0.44	0.19	0.37	0.57	0.17	0.16
*P*	>0.05	>0.05	>0.05	>0.05	>0.05	>0.05	>0.05	>0.05	>0.05

*F is the test result.*

The reason for little difference in the psychological status of teachers with different education levels may be that teachers with different educational levels have different expectations. They have different academic skills in competition. For example, regarding the application of teachers in promoting vocational qualifications, colleges have different educational requirements for different vocational qualifications. Doctors have psychological problems because of the high expectation of academics.

### Comparison of the Psychological Status of Teachers With Different Titles: Junior, Intermediate, Deputy Senior, and Senior

The results of SCL-90 (Table 5) reveal that besides somatization, senior teachers score higher than middle-level teachers in interpersonal sensitivity and other factors. The level of obsessive-compulsive symptoms, interpersonal sensitivity, depression, anxiety, and mental symptoms of deputy senior teachers is higher than that of other teachers. However, there is no significant difference in factor scores among teachers with different professional titles.

**TABLE 5:** Comparison of survey results of teachers with different professional titles.

	Somatization	Obsession	Interpersonal sensitivity	Depression	Anxiety	Hostility	Terror	Paranoia	Other
Junior	1.81 ± 0.80	2.1 ± 0.85	1.79 ± 0.88	1.85 ± 0.72	1.77 ± 0.81	1.80 ± 0.84	1.64 ± 0.84	1.82 ± 0.83	1.67 ± 0.74
Intermediate	1.79 ± 0.72	2.05 ± 0.73	1.75 ± 0.72	1.90 ± 0.72	1.72 ± 0.73	1.53 ± 0.73	1.54 ± 0.73	1.75 ± 0.75	1.76 ± 0.73
Deputy senior	1.74 ± 0.67	2.03 ± 0.72	1.75 ± 0.70	1.86 ± 0.75	1.70 ± 0.71	1.73 ± 0.71	1.50 ± 0.68	1.72 ± 0.71	1.60 ± 0.73
Senior	1.90 ± 0.70	2.3 ± 1.82	1.87 ± 0.74	1.99 ± 0.71	1.82 ± 0.76	1.80 ± 0.71	1.56 ± 0.74	1.79 ± 0.73	1.68 ± 0.70
*F*	0.50	0.77	0.40	0.44	0.19	0.37	0.57	0.17	0.16
*P*	>0.05	>0.05	>0.05	>0.05	>0.05	>0.05	>0.05	>0.05	>0.05

It is believed that there is no significant difference in the psychological status of teachers with different professional titles, which is closely related to the reform of the professional title evaluation system. Colleges with high standard professional title promotion require teachers with different professional titles to have a certain amount of documentation and writing related to education, teaching and scientific research promotion and other related professional courses. This undoubtedly brings certain pressure to teachers of different titles and ages.

### Comparison of the Psychological Status of Teachers in Different Disciplines

**FIGURE 4:**
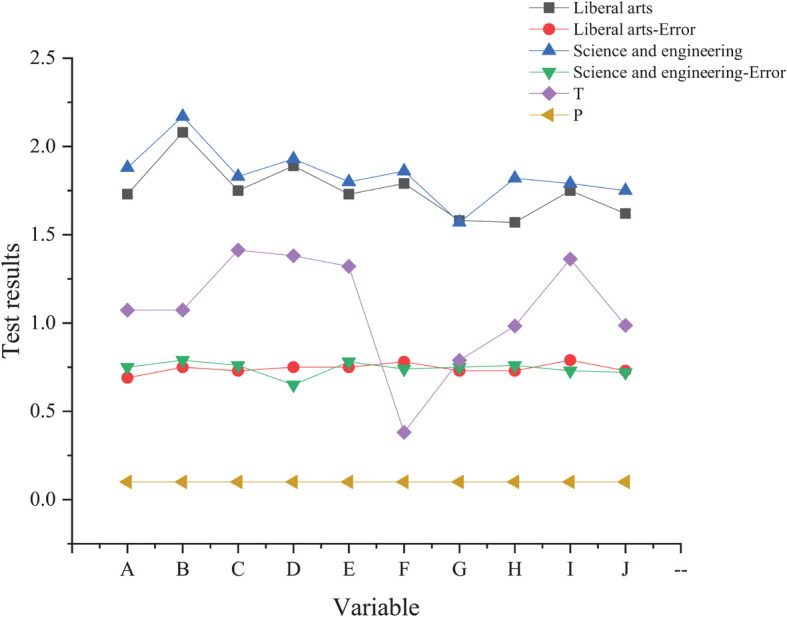
Comparison of survey results of teachers in different disciplines.

Teachers are divided into two categories: teachers engaged in scientific and engineering education research and teachers engaged in liberal arts education research. The results of SCL-90 (Figure 4) prove that the scores of teachers engaged in teaching and scientific research and engineering are higher than those of liberal arts teachers engaged in teaching and research, while the difference is not significant.

The reason for little difference in the psychological status of teachers between different disciplines may be the integration of modern disciplines, and fuzzy boundaries, showing a trend of cross integration. With the coming of the knowledge economy, science, engineering, and art teachers are required to have educational methods and contents suitable for the development of the times, so as to match and adapt to the new situation of the current higher education reform. Teachers engaged in science, engineering, and art should conduct teaching and carry out scientific research, which brings about great work pressure.

### Comparison of the Psychological Status of Teachers of Different Teaching Ages

Teachers are divided into five groups according to the different teaching years: 1–5 years, 6–10 years, 11–15 years, 16–20 years, and above. The results of SCL-90 (Table 6) reveal that the factor scores of teachers teaching for 11–15 years are higher than those of other teachers, while the difference is not significant.

**TABLE 6:** Comparison of survey results of teachers with different teaching years.

	Somatization	Obsession	Interpersonal sensitivity	Depression	Anxiety	Hostility	Terror	Paranoia	Other
1–5 years	1.87 ± 0.78	1.75 ± 0.76	1.86 ± 0.76	1.76 ± 0.81	1.78 ± 0.91	1.54 ± 0.77	1.80 ± 0.80	1.77 ± 0.80	1.78 ± 0.37
6–10 years	1.74 ± 0.67	2.03 ± 0.72	1.76 ± 0.70	1.86 ± 0.75	1.70 ± 0.71	1.76 ± 0.71	1.50 ± 0.68	1.72 ± 0.71	1.60 ± 0.73
11–15 years	1.90 ± 0.70	2.3 ± 1.83	1.87 ± 0.74	1.99 ± 0.71	1.82 ± 0.76	1.80 ± 0.71	1.46 ± 0.74	1.79 ± 0.73	1.69 ± 0.70
16–20 years	1.90 ± 0.80	2.11 ± 0.82	1.88 ± 0.78	1.98 ± 0.77	1.86 ± 0.85	1.83 ± 0.83	1.67 ± 0.86	1.81 ± 0.80	1.77 ± 0.83
Over 20 years	1.85 ± 0.76	2.01 ± 0.75	1.71 ± 0.75	1.83 ± 0.76	1.68 ± 0.78	1.69 ± 0.70	1.47 ± 0.66	1.76 ± 0.74	1.74 ± 0.71
*F*	0.50	0.77	0.40	0.44	0.19	0.37	0.57	0.17	0.16
*P*	>0.05	>0.05	>0.05	>0.05	>0.05	>0.05	>0.05	>0.05	>0.05

Table 6 reveals that there is no significant difference in the psychological level of teachers in different grades, because the continuous development of modern education technology requires teachers of different ages to change the traditional educational thought, educational concept, and method education, and establish the concept of lifelong learning. At present, the promotion of higher education reform requires teachers of different grades to improve their abilities constantly, making them invincible in competitive colleges.

## Discussion

### The Implications for Academic Research

For a long time, the research of AI focused on the improvement of algorithms and models step by step, Carroll and Dahlstrom (2021) pointed out focusing on the technical content based on prediction ability, while the attention paid to interpretation ability was slightly insufficient. At present, most HCI studies in the world focus on the technical level, ignoring the social level and psychological factors of HCI. However, the success of technology does not mean the success of products and the harmony of the man-machine relationship. Only when the application of technology adapts to human nature and human development, can it be gradually accepted and developed by the world. Present HCI is far from reaching the ideal degree of natural interaction. More and more in-depth research is needed on people’s own mental model and people’s understanding of the HCI process itself. This psychological model includes not only people’s understanding of the operation mode of the system itself, but also people’s understanding of how the operation mode affects their cognition and emotion. In interaction design, it is essential to adhere to the human-centered design principle and understand the human information processing process and its operation mechanism, which is of great value for HCI. Compared with previous studies, this exploration obtains the other assistance system to improve the mental health level of college teachers, strengthen the mental health education of teachers, implement a reasonable and effective incentive mechanism, further improve the performance appraisal system of colleges, and further improve the teacher income distribution system. Monea (2020) suggested that the interaction between human and AI is gradually deepening, and HCI is being used in the occasion of multi task cooperation. Therefore, the interpretability of AI has become a necessary factor to achieve full human–computer cooperation.

### The Implications for Industry and Practices

With the continuous improvement of the autonomy of machines, AI such as Intel’s adaptive robot has realized the ability of autonomous interaction and repair. How to make the machine take the initiative to educate, so that users can fully trust and understand AI and participate in interaction, puts forward higher requirements for the interpretability of the system. Moreover, the management of colleges should be people-oriented. Due to time constraints, the occupational mental health status of college teachers is only investigated for some time, and it fails to track the dynamic changes of occupational mental health status in real-time. Based on the analysis of the causes and the discussion of teachers’ psychological problems, measures are taken to improve their mental health education and mental health status, and empirical research is carried out. Studying the professional pressure and mental health problems of college teachers has crucial practical significance for alleviating the professional pressure of college teachers and improving their mental health level. However, due to the limited SCL-90 item scale, the analysis results of the changes in the mental health level of teaching staff need to be further verified. In the later research, the samples will continue to be expanded and analyzed, and more factors will be included in the discussion. Zhu (2019) discussed the educational reform being caused by AI technology, the reasons why educational reform needs technical support, and how to use technology to promote educational reform, suggesting that the main research field is how technology affects educational reform. This exploration is to take the teaching staff in academic management of colleges as the research object, and explore the evolution process of college teachers’ psychological change by studying from the perspectives of age, professional title, education background, discipline and teaching year. Therefore, relatively speaking, the research content of this exploration is more in-depth and comprehensive.

## Conclusion

The theory of teaching staffs’ psychological status, and the characteristics of staff in academic management of colleges are analyzed under the background of AI and HCI. Then, the psychological changes of staff under different conditions are analyzed through the method of questionnaire and model test. The results show that the mental health level of college teachers is lower than that of the national adult norm; the mental health level of female teachers is higher than that of male teachers; there is no significant difference in the mental health status of college teachers with different ages, professional titles, education backgrounds, disciplines, and teaching years; there is a significant correlation between professional academic and mental health, indicating a significant impact of teachers’ professional academic on teachers’ mental health. The research still has limitations. The research is based on the evaluation practice of AI on interactive environment management, ignoring the evaluation of interpretation quality. Meantime, the design based on evaluation from the perspective of developers also has some limitations in index selection. In the future research, a multi-level and multi-angle evaluation system will be built from the perspectives of AI itself, developers, and users. In short, for local colleges and universities, the mental health problems of teaching staff cannot be ignored. A healthy and positive mental state is not only conducive to the improvement of teachers’ academic level and teaching ability, but also promote the high-quality development of the school.

## Data Availability Statement

The raw data supporting the conclusions of this article will be made available by the authors, without undue reservation.

## Ethics Statement

The studies involving human participants were reviewed and approved by the Northeast Normal University Ethics Committee. The patients/participants provided their written informed consent to participate in this study. Written informed consent was obtained from the individual(s) for the publication of any potentially identifiable images or data included in this article.

## Author Contributions

HG: conceptualization and writing – original draft. QC: software and resources. SH: methodology. BZ: data curation and supervision. All authors listed have made a substantial, direct and intellectual contribution to the work, and approved it for publication.

## Conflict of Interest

The authors declare that the research was conducted in the absence of any commercial or financial relationships that could be construed as a potential conflict of interest.

## Publisher’s Note

All claims expressed in this article are solely those of the authors and do not necessarily represent those of their affiliated organizations, or those of the publisher, the editors and the reviewers. Any product that may be evaluated in this article, or claim that may be made by its manufacturer, is not guaranteed or endorsed by the publisher.
