# Supplementary material for: The Influence of “Artificial Intelligence + Human–Computer Interaction” on Teachers’ Psychological Changes in Academic Management in Colleges
Source: Front Psychol. 2021 Nov 19;12:730345. doi: 10.3389/fpsyg.2021.730345 (PMC8639499; doi:10.3389/fpsyg.2021.730345)
Supplement: Supplementary file 1 [file Table_1.docx]

**Appendix**

**Questionnaire on psychological changes of college staff**

1. Basic information

(1) Your gender A. Male B. Female

(2) Your age A. Under 35 years old B. Under 50 years old C. Over 51 years old

(3) Educational level A. Below junior college B. Undergraduate C. Master D. Doctor

(4) Current title A. Junior B. Intermediate C. Deputy senior D. Senior

(5) What subject do you teach? A. Science and engineering B. Liberal arts

(6) How many years have you worked? A. 1-10 years B. 11-20 years C. Over 20 years

2. Related problems

(1) Do you feel pressure to work as a teacher? A. No pressure B. Some pressure C. Great pressure

(2) If work pressure is one of your sources of pressure, which of the following aspects make you feel stressed? A. Heavy scientific research task B. High teaching requirements C. Difficulty in obtaining projects D. Title evaluation E. Post assessment F. Unclear development objectives G. Difficult publication of academic achievements H. Poor teaching and research conditions I. Others

(3) What aspects of the school system do you feel pressure on? A. Personnel distribution system B. Professional title evaluation system C. Performance appraisal system

(4) Do you feel that you are often despised and criticized by your leaders at work? A. Often B. Sometimes C. Never

(5) Do you feel that you don't get along well with your colleagues and there are all kinds of barriers? A. Often B. Sometimes C. Never

(6) What do you think of the expectations of parents and Society for teachers? A. Very high B. High C. Not high

(7) Do you often feel bored? A. Often B. Sometimes C. Never

(8) Do you talk to yourself alone? A. Often B. Sometimes C. Never

(9) Have you ever thought of suicide because of pessimism in life? A. Often B. Sometimes C. Never

(10) Have you ever been unable to sleep at night because of work problems? A. Often B. Sometimes C. Never

(11) Are you worried about your job stability? A. Never worried B. Occasionally worried C. Often worried

(12) If possible, would you like to change your job? A. I don't want to. B. I don't want to change my career, but I want to find a better position in this industry. C. I really want to change my job

(13) How do you feel about the work, teaching and scientific research environment of your department? A. Very satisfied B. Satisfied C. Unable to explain clearly D. Not very satisfied E. Very dissatisfied

(14) How do you feel about your relationship with students? A. Very satisfied B. Satisfied C. Unable to explain clearly D. Not very satisfied E. Very dissatisfied

(15) How do you feel about the colleague relationship in the school? A. Very satisfied B. Satisfied C. Unable to explain clearly D. Not very satisfied E. Very dissatisfied

(16) How do you feel about the leadership of your department? A. Very satisfied B. Satisfied C. Unable to explain clearly D. Not very satisfied E. Very dissatisfied

(17) What do you think of school management? A. Very strict B. relatively strict C. generally D. relatively loose E. very loose

(18) How do you feel about your school's teaching evaluation system? A. Very satisfied B. Satisfied C. Unable to explain clearly D. Not very satisfied E. Very dissatisfied

(19) How do you feel about the current form of teacher training? A. Very satisfied B. Satisfied C. Unable to explain clearly D. Not very satisfied E. Very dissatisfied

(20) How do you feel about the current professional title evaluation form? A. Very satisfied B. Satisfied C. Unable to explain clearly D. Not very satisfied E. Very dissatisfied
